# Industrial Pollution and Health in Louisiana: A Systematic Review of Quantitative and Qualitative Studies

**DOI:** 10.1007/s40572-026-00540-y

**Published:** 2026-05-22

**Authors:** Miriam R. Simon, Juliet M. Nussbaum, Kenya Goodson, Barbara L. Allen, Michelle Smith, Isabella U. Yalif, Alison K. Cohen

**Affiliations:** 1 School of Medicine, University of California San Francisco, San Francisco, CA USA; 2 Center for Health Professions & Health Concerns, Dillard University, New Orleans, LA USA; 3 Department of Science, Technology, & Society, Virginia Tech, Blacksburg, VA USA; 4 Minority Health & Health Equity Research Center, Dillard University, New Orleans, LA USA; 5 Vanderbilt University, Nashville, TN USA; 6 Department of Epidemiology & Biostatistics and Philip R. Lee Institute for Health Policy Studies, School of Medicine, University of California San Francisco, San Francisco, CA USA

**Keywords:** Environmental health, Louisiana, Environmental pollution, Industrial pollution, Health outcomes, Systematic review

## Abstract

**Purpose of Review:**

Louisiana has one of the largest concentrations of petrochemical industry in the USA. Many studies have assessed patterns of industrial pollution and health in Louisiana; we aim to systematically review this evidence.

We systematically searched PubMed, Web of Science, Embase, APA PsycInfo, and GreenFILE for peer-reviewed papers published 1999–2024 that reported geographical variation in health or industrial pollution, and/or tested for an association between the two in Louisiana. We used Covidence to support standardized review and extraction.

**Recent Findings:**

We identified 2485 non-duplicate papers in our search; 53 met the inclusion criteria. Most reported quantitative findings. All studies of industrial pollution described air pollution (some also described other pollution). Studies described various health outcomes, including cancer, respiratory health, mortality, and COVID-19. Overall, people who lived closer to industrial activity had higher pollution exposure and worse health. Black and lower-income residents were exposed to more industrial activity than white and higher-income residents. Twenty-one studies assessed statistical associations between industrial pollution and health; many found an association. Twenty-one studies were quantitative and adjusted for confounding, 29 studies did not adjust for confounding (including qualitative studies), and three studies did not adjust for confounding and had authors with industry ties.

**Summary:**

Evidence suggests that there is a higher burden of air pollution and worse health outcomes in industrialized areas of Louisiana. While there was some evidence of significant associations between industrial pollution and health outcomes, research with larger sample sizes and improved pollution exposure measurements could be informative.

## Introduction

### Industrial Pollution and Health Outcomes Worldwide

A substantial body of evidence from around the world has found that industrial pollution of the environment is linked to a wide variety of health outcomes [1–3], including mortality, cardiovascular disease, respiratory disease, and malignancies [4–6]. These associations have been observed across many diverse settings and contexts [7]. Airborne pollutants from industrial sources, including fine particulate matter (PM_2.5_), nitrogen oxides (NO_x_), sulfur oxides (SO_x_), and volatile organic compounds (VOCs), are among the most well-studied exposures, with strong links to several health outcomes [8–10]. For example, long-term exposure to PM_2.5_ has been linked to increased risk of cardiovascular disease while community and occupational exposures near industrial sites have been linked to elevated cancer rates [7, 11]. Industrial contamination of soil and water have been associated with exposure to heavy metals and other toxic substances [12, 13], which accumulate in the body over time and can contribute to disease risk [14]. Specifically, exposure to arsenic, lead and cadmium through contaminated soil and water has been linked to cardiovascular, renal, and neurological outcomes [15].

### Environmental Exposure Inequities in the United States

The burden of industrial pollution is not evenly distributed. There is substantial documentation of inequities in exposures to environmental pollution in the United States of America (USA), particularly for people from historically marginalized groups [16–19]. Existing systematic reviews consistently show that lower socioeconomic position (SEP) communities face higher exposure to air pollutants [20–22] and are more likely to live in fenceline communities closer to industrial sources of pollution [23]. Nationwide analyses have shown that Black and Hispanic populations in the USA are exposed to higher levels of PM_2.5_ than white populations even after accounting for income differences [24]. These uneven patterns of exposure are often shaped by historical patterns of land use, zoning, and structural inequities [25–27]. Higher concentrations of air pollution have been linked to formerly redlined neighborhoods underscoring how discriminatory housing policies affect communities decades after they were enacted [28]. Thus, industrial pollution is a major contributor to health disparities in the USA and worldwide.

### Louisiana as a Major Petrochemical Hub

From the 1960 s to today, the state of Louisiana in the USA has been the site of extensive growth in the petrochemical industry [29, 30] and currently has one of the largest concentrations of petrochemical industrial activity in the USA [31]. The 85-mile stretch of the Mississippi River between Baton Rouge and New Orleans, is currently home to over 200 petrochemical plants and allied industries [32]. Many more facilities are located on the southwestern part of Louisiana, near the Texas border, and many oil refineries dot the landscape in the northern part of the state [33]. Additionally, the petrochemical industry has been rapidly expanding and is projected for further growth [29].

### Historical Roots of Environmental Injustice in Louisiana

Louisiana provides a case study of the state-industry alliances that have produced some of the USA’s most emblematic environmental justice (EJ) controversies and communities [30, 34–36]. Much of the land industry occupies in the Mississippi River Industrial Corridor was former plantations sold to corporations for development. Directly adjacent to these large properties were smaller homesteads deeded to former enslaved people in extended family groups immediately following the Civil War. In many cases, their descendants still inhabit these properties, leading to the communities at the fenceline of many of this area’s large industrial sites being majority poor and Black [30]. Many residents refer to this area as “Cancer Alley”, reflecting the connections that residents have long drawn between the environmental injustices they experience from industry and the health inequities they observe in their communities. Although Cancer Alley is the most well-known industrial region in the state, there are many other industrial zones across Louisiana where local residents have struggled for a cleaner environment and responsible industrial regulations, as evidenced by the 40 + groups and sites included in the community atlas of the non-profit Louisiana Environmental Action Network [37].

While the focus of our systematic review is on the health impact of industrial pollution in the state, there is also a long history of health impacts from hurricanes (e.g. Hurricane Katrina, Hurricane Rita) [38, 39] and other events like the Deepwater Horizon oil spill [40].

### Why a Systematic Review is Needed in Louisiana

Given the context of disproportionate environmental pollution and the history of research and environmental justice concerns and advocacy in the state, there is an opportunity to summarize the body of knowledge about industrial pollution and health in the region. The extensive quantitative and qualitative research findings from Louisiana may be generalizable to other industrialized regions, including illustrating how different types of studies in the same area can provide complementary insights.

### Knowledge Gap and Study Objective

To the best of our knowledge, there have been no systematic reviews of the patterns of pollution exposure and health outcomes, as well as the association between the two, focusing specifically on Louisiana.

This systematic review aims to synthesize the body of knowledge on industrial pollution and health in Louisiana, aiming to contribute to a deeper understanding of the interplay of these issues in the region.

## Methods

### Inclusion and Exclusion Criteria

We included peer-reviewed studies published from 1999 through 2024 in academic journals that either (1) describe (quantitatively and/or qualitatively) patterns of residential exposure to industrial environmental pollution in Louisiana, (2) describe (quantitatively or qualitatively) geographic patterns of human health outcomes in Louisiana, or (3) assess relationships between residential exposure to industrial pollution and human health in Louisiana. We specifically focused on residential exposure to chemical and petrochemical industrial pollution in our review; we did not focus on research regarding other sources of pollution, such as agricultural or traffic pollution. We restricted pollution media to air, water, and soil. All study types (e.g., observational, experimental, ethnographic) were eligible for inclusion, as well as all health outcomes. We excluded research that focused on occupational exposures. While we did not initially exclude any articles based on language, all relevant articles were published in English.

To minimize the risk of bias in our review, we exclusively included peer-reviewed articles. We excluded abstracts, commentaries, books and book chapters, and non-peer reviewed studies. However, we acknowledge that this leaves our review susceptible to publication bias, which suggests that statistically significant results (for quantitative papers) may be more likely to be published [41]. We also excluded any study that did not meet the inclusion criteria.

Quantitative and qualitative studies that examine pollution and/or its effects on human health can be found in a variety of journals, including those related to medicine and public health, environmental studies and sciences, and sociology and anthropology. We systematically searched the following electronic databases: PubMed, Web of Science, Embase, APA PsycInfo, and GreenFILE. All searches were conducted from July 3–11, 2024. The dates and search strategy for each database can be found in Table 1. Author 1 performed the database searches with the guidance of an information specialist. If the databases included a “peer reviewed” search filter, it was utilized. In the Web of Science database, using “Louisiana” as a search term resulted in many papers that had authors with Louisiana-based institution affiliations, but did not report on findings related to Louisiana; for this reason, the Web of Science search was limited to our search terms appearing in the title and/or abstract.

**Table 1:** Database-specific search strategies used in the systematic review

Database	Search terms	Special filters	Number of articles	Date of search
PubMed	("Louisiana") AND (Environmental Justice[Mesh] OR "environmental justice" OR "environmental health" OR "Environmental Health"[Mesh] OR "environmental injustice*" OR "environmental risk*" OR "environmental racism" OR "Air Pollution"[Mesh] OR "Water Pollution"[Mesh] OR "Petroleum Pollution"[Mesh] OR "Environmental Pollution"[Mesh] OR "pollut*" OR "Environmental Exposure"[Mesh] OR "environmental exposure*" OR "environmental hazard*" OR "environmental impact*" OR "Hazardous Waste"[Mesh] OR "hazardous waste" OR "Pesticide Residues"[Mesh] OR "pesticide*" OR "Oil and Gas Industry"[Mesh] OR "refiner*" OR "Plastics"[Mesh] OR "plastic*" OR “Petroleum”[Mesh] OR "petrochemical*" OR "cancer alley" OR "chemical corridor" OR "chemical plant" OR "chemicals" OR "Manufacturing and Industrial Facilities"[Mesh] OR "industrial plant*" OR “petroleum” OR “oil spill” OR "Pesticide Residues"[Mesh] OR “fertilizer*”) AND ("health" OR “neoplasms”[Mesh] OR "cancer*" OR "malignan*" OR "tumor*" OR "respiratory"OR "lung related disease*" OR "Lung Diseases"[Mesh] OR "lung disease*" OR "eye irritation" OR "asthma" OR "Chronic Disease"[Mesh] OR "chronic illness*" OR “chronic disease*” OR "Reproductive Health"[Mesh] OR "reproductive health" OR "Mortality"[Mesh] "mortalit*" OR "Mental Health"[Mesh] OR "mental health")	Date range 1999 - 2024	184	July 3, 2024
Web of Science	("Louisiana") AND ("environmental justice" OR "environmental health" OR "environmental injustice*" OR "environmental risk*" OR "environmental racism" OR "pollut*" OR "environmental exposure*" OR "environmental hazard*" OR "environmental impact*" OR "hazardous waste" OR "pesticide*" OR "refiner*" OR "plastic*" OR "petrochemical*" OR "cancer alley" OR "chemical corridor" OR "chemical plant" OR "chemicals" OR "industrial plant*" OR “petroleum” OR “fertilizer*” OR “oil spill”) AND ("health" OR "cancer*" OR "malignan*" OR "tumor*" OR "respiratory” OR "lung related disease*" OR "lung disease*" OR "eye irritation" OR "asthma" OR "chronic illness*" OR “chronic disease*” OR "reproductive health" OR "mortalit*" OR "mental health") (topic)	Date range 1999 - 2024	368	July 3, 2024
GreenFILE	("Louisiana") AND ("environmental justice" OR "environmental health" OR "environmental injustice*" OR "environmental risk*" OR "environmental racism" OR "pollut*" OR "environmental exposure*" OR "environmental hazard*" OR "environmental impact*" OR "hazardous waste" OR "pesticide*" OR "refiner*" OR "plastic*" OR "petrochemical*" OR "cancer alley" OR "chemical corridor" OR "chemical plant" OR "chemicals" OR "industrial plant*" OR “petroleum” OR “fertilizer*” OR “oil spill”) AND ("health" OR "cancer*" OR "malignan*" OR "tumor*" OR "respiratory” OR "lung related disease*" OR "lung disease*" OR "eye irritation" OR "asthma" OR "chronic illness*" OR “chronic disease*” OR "reproductive health" OR "mortalit*" OR "mental health")	Date range 1999 - 2024 Peer reviewed	385	July 5, 2024
APA Psycinfo	("Louisiana") AND ("environmental justice" OR "environmental health" OR "environmental injustice*" OR "environmental risk*" OR "environmental racism" OR "pollut*" OR "environmental exposure*" OR "environmental hazard*" OR "environmental impact*" OR "hazardous waste" OR "pesticide*" OR "refiner*" OR "plastic*" OR "petrochemical*" OR "cancer alley" OR "chemical corridor" OR "chemical plant" OR "chemicals" OR "industrial plant*" OR “petroleum” OR “fertilizer*” OR “oil spill”) AND ("health" OR "cancer*" OR "malignan*" OR "tumor*" OR "respiratory” OR "lung related disease*" OR "lung disease*" OR "eye irritation" OR "asthma" OR "chronic illness*" OR “chronic disease*” OR "reproductive health" OR "mortalit*" OR "mental health")		132	July 8, 2024
Embase	("Louisiana") AND ("environmental justice" OR "environmental health" OR "environmental injustice*" OR "environmental risk*" OR "environmental racism" OR "pollut*" OR "environmental exposure*" OR "environmental hazard*" OR "environmental impact*" OR "hazardous waste" OR "pesticide*" OR "refiner*" OR "plastic*" OR "petrochemical*" OR "cancer alley" OR "chemical corridor" OR "chemical plant" OR "chemicals" OR "industrial plant*" OR “petroleum” OR “fertilizer*” OR “oil spill”) AND ("health" OR "cancer*" OR "malignan*" OR "tumor*" OR "respiratory” OR "lung related disease*" OR "lung disease*" OR "eye irritation" OR "asthma" OR "chronic illness*" OR “chronic disease*” OR "reproductive health" OR "mortalit*" OR "mental health")		2040	July 11, 2024

**Fig. 1:**
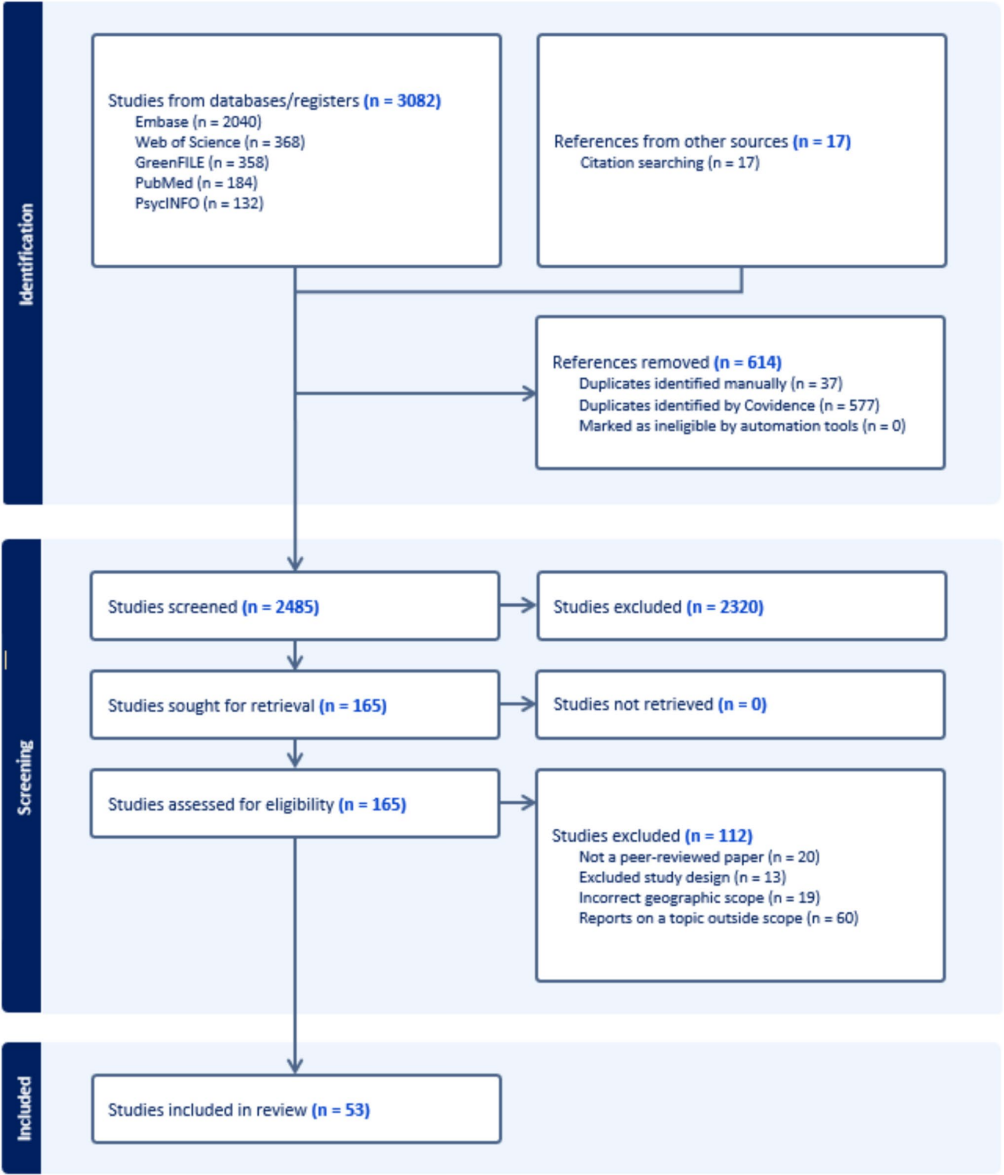
PRISMA (Preferred Reporting Items for Systematic reviews and Meta-Analyses) flow diagram depicting the process of study identification, screening, eligibility, and final inclusion for the systematic review, including reasons for study exclusion

We imported search results from each database into Covidence, a software platform that helps streamline the process of conducting a systematic review. Figure 1 shows the PRISMA (Preferred Reporting Items for Systematic reviews and Meta-Analyses) flow diagram of inclusion and exclusion. PRISMA is a widely-recognized and utilized set of guidelines for systematically reporting on systematic reviews in a consistently clear way [42]. In the identification stage, references imported from the databases listed in Table 1 and studies retrieved from citation searching were screened for duplicates. References then advanced to the screening stage, where studies were screened by their title and abstract for eligibility using predetermined inclusion criteria. Studies for which eligibility could not be determined based on title and abstract, were advanced to full text review to ensure thorough evaluation. In full text review, papers were excluded for incorrect topic, inappropriate study design, irrelevant location, or lack of peer review. Authors 1, 2, 6, and 7 did title and abstract and full text review screening; Authors 1, 2, 3, and 7 conducted data extraction from the included full texts using a standardized data extraction form in Covidence.

### Data Extraction

We created a data extraction form in Covidence to systematically collect relevant information for each included study. For qualitative studies, we categorized reports of chemical odors and smoke plumes from industrial facilities as presence of air pollution. Water pollution was identified through descriptions of specific visual observations, such as orange foam and plastic beads in water [43], or through accounts of known water pollution events reported by residents [44]. Many qualitative studies included accounts of observed changes to vegetation (i.e. discolored/dying plants, inability of plants to grow) or wildlife; this could be evidence of air, water, and/or soil pollution, so we did not categorize these.

Each study was assessed by at least two authors: one conducted an initial review and collected key attributes and findings in Covidence, while the other re-read the study and cross-checked the data to ensure accuracy and consistency.

A meta-analysis was not possible due to the heterogeneity of studies; instead, we describe key findings from each paper and synthesize across papers qualitatively.

### Methodological Quality Assessment

Given the heterogeneity of the research reviewed, including both quantitative and qualitative studies, there is not an established tool that is a good fit for assessing the quality of the studies. Instead, we developed a simple scoring system to assess study quality which included domains for confounding adjustment and author petrochemical industry ties. Studies received a score of 1 if they adjusted for confounding using multivariable statistical analysis or causal inference methods ((e.g., regression, propensity score, or mediation analysis). Studies that did not adjust for confounders received a score of 0. Studies received a score of −1 if the authors had industry ties and did not adjust for confounding. (Of note, no studies with authors with industry ties adjusted for confounding.) Not all domains were applicable to all study designs; qualitative studies are not designed to be adjusted for confounding and have therefore received a score of 0. While this is a simplification, readers can consider scores of 0 and −1 to indicate lower quality and scores of 1 to indicate higher quality.

In consideration of our limited ability to assess study quality, only peer reviewed research was included in this systematic review.

## Results

Our search identified 3,099 records, 2,485 of which were non-duplicate records. Removal of duplicate results and title and abstract screening resulted in 2,320 records excluded and 165 peer-reviewed papers advancing to the full-text review stage. After a full text review, 53 papers were included in our final sample. Records were excluded at the full-text review stage for several reasons (Fig. 1); where records had multiple reasons for exclusion, we assigned the first criteria met in the list. Twenty records were not peer-reviewed papers, including 10 conference abstracts, reports, or posters, 3 news articles, a book chapter, a graduate thesis, a pre-printed article, an editorial article, a manuscript critique, an essay, and a study protocol. Thirteen papers had an excluded study design, including 8 literature reviews, 2 commentaries, a framework paper, a consensus article, and a simulation paper. Papers were also excluded for having an incorrect geographic scope (*n* = 19), including 11 papers that did not report on data from Louisiana residents, 4 that aggregated Louisiana resident data with that of other states, and 4 that reported on Louisiana data with no assessment of variation of health or pollution data within the state (i.e., statewide estimates only). Finally, 60 papers reported on topics outside the scope of our current review, including studies of the effects of Hurricane Katrina (*n* = 12), the Deepwater Horizon spill (*n* = 21), and other environmental disasters (*n* = 10), which merit separate investigation. Others examined the environmental distribution or health impacts of heavy metals (*n* = 2), which can arise from both industrial and non-industrial sources [45]. We also excluded studies that did not present data related to residential exposures to industrial pollution (*n* = 15).

The characteristics of the included studies are presented in Table 2. Of the 53 included studies, 44 were quantitative, 7 were qualitative, and 2 were multi-method studies that utilized both qualitative and quantitative data. Over half of the quantitative studies had an ecological design (*n* = 25), where data were aggregated at the census tract, ZIP code, or parish (county)-level; the rest were cross-sectional studies (*n* = 6), cohort studies (*n* = 2), and case–control studies (*n* = 2). Of the quantitative and multi-method studies, 11 presented data about patterns of industrial pollution only, 6 presented data on health outcomes only, 8 presented on patterns of both industrial pollution and health, and 19 studies assessed statistical associations between industrial pollution and health outcomes. All qualitative studies described links between industrial pollution and health.

**Table 2:** Extracted study characteristics, exposures, and outcomes from studies included in the systematic review

Study ID	Study type^a^	Year(s) of data	Sample size^b^	Location in Louisiana^c^	Measure(s) of pollution exposure^d,e^	Measure(s) of health^f^	Study assessment score^g^
Adeola [46]	Quantitative (Cross-sectional)	1996	110 residents	New Orleans Metropolitan Area	Residence in an EPA National Priorities List (NPL) site vs. non-NPL site	Allergic conditions, cancer, dermatologic conditions, eye conditions, gastrointestinal disorders, general/constitutional symptoms, hearing condition, mental health, reproductive health, respiratory conditions	0
Bakshi et al. [47]	Quantitative (Ecological)	2014–2021	1035 census tracts	Statewide	Census tract level NATA respiratory hazard index; average annual PM2.5; average summer ozone levels	Asthma, COVID-19	1
Bera and Hrybyk [48]	Quantitative (Cross-sectional)	2012, 2013	128 residents (Belle Chasse), 103 (Norco), 132 (Chalmette)	Belle Chasse, Plaquemines Parish; Norco, St. Charles Parish; Chalmette, St. Bernard Parish	Accidental chemical release events from industrial facilities	Eye conditions, general/constitutional symptoms, respiratory conditions	0
Berry [49]	Qualitative	1991–2001	Not provided	Convent, St. James Parish	Community member and local activists' lived experiences and perceptions of industrial pollution	Asthma	0
Billings [50]	Quantitative (Cross-sectional)	1997–2001	Not provided	Statewide	Residence in seven parishes in "the Industrial Corridor" (East Baton Rouge, West Baton Rouge, Ascension, Iberville, St. James, St. Charles, St. John the Baptist) vs. others in LA	Cancer	−1
Blanks et al. [51]	Quantitative (Cross-sectional)	2019–2020	101 cemeteries	Ascension Parish, St. James Parish	Proximity of cemeteries to industrial facilities	n/a	0
Blodgett [52]	Quantitative (Ecological)	1987–2002	21,216 residents	St. James Parish	Location of and total emitted pollution from manufacturing facilities (LDEQ data, 1987–2002)	n/a	0
Boopathy et al. [53]	Quantitative (Cross-sectional)	1998 - 1999	6,498 hospital visits	Houma, Terrebonne Parish	n/a	Asthma	0
Burby [54]	Quantitative (Cross-sectional)	1996	399 residents	Baton Rouge	Distance to and number of TRI reporting facilities within 3 miles of home; pounds of toxic chemicals released to the air by TRI-reporting facilities within 3 miles of home	Overall health	1
Carroll et al. [55]	Quantitative (Ecological)	2000–2013	48,551 women	Statewide	Parish-level modeled estimates of 2005 EPA chemical emissions variables	Cancer	1
Dasgupta et al. [56]	Quantitative (Ecological)	2021	64 parishes	Statewide	Residence in Cancer Alley parishes vs. others in LA	Asthma, cancer, COVID-19, respiratory conditions	0
Davies [44]	Qualitative	2015–2018	25 residents and activists	St. James Parish	Community member lived experiences and perceptions of industrial pollution	Cancer, mortality, overall health, respiratory conditions	0
Davies [57]	Qualitative	2016–2018	Not provided	Freetown, St. James Parish	Community member lived experiences and perceptions of industrial pollution	Cancer, cardiovascular disease, dermatologic conditions, metabolic conditions, neurologic/neurodevelopmental conditions, respiratory conditions	0
Fos et al. [58]	Quantitative (Ecological)	2019	1,635,750 residents	Statewide	Residence in 11 Cancer Alley parishes (Ascension, East Baton Rouge, Iberville, Jefferson, Orleans, Plaquemines, St. Bernard, St. Charles, St. James, St. John the Baptist, and West Baton Rouge parishes) vs. others in LA	COVID-19	0
Fos et al. [59]	Quantitative (Ecological)	2017, 2019	64 Louisiana Parishes 4 cities/counties in the U.S The U.S	Statewide	Residence in 11 Cancer Alley parishes (Ascension, East Baton Rouge, Iberville, Jefferson, Orleans, Plaquemines, St. Bernard, St. Charles, St. James, St. John the Baptist, and West Baton Rouge Parishes) vs. others in LA	COVID-19, general/constitutional symptoms, mental health, metabolic conditions, mortality	0
Holden and Ratard [60]	Quantitative (Cross-sectional)	2005–2009	64 parishes	Livonia Fordoche, Mossville, Coteau, New Orleans, Statewide	n/a	Cancer	0
Hu et al. [61]	Quantitative (Ecological)	2014–2020	64 parishes	Statewide	Average daily density of PM2.5, number of TRI sites, pounds of toxic chemical release, instances of drinking water violations	COVID-19	1
Hugh-Jones et al. [62]	Quantitative (Ecological)	1995–2014	23,224 residents with Parkinson's disease	Statewide	n/a	Neurologic/neurodevelopmental conditions	1
Hunt et al. [63]	Quantitative (Cross-sectional)	Not specified	116 residents with lung cancer	11 Cancer Alley parishes (Ascension, East Baton Rouge, Iberville, Jefferson, Orleans, Plaquemines, St. Bernard, St. Charles, St. John the Baptist, St. James, and West Baton Rouge parishes)	n/a	Cancer	0
James et al. [64]	Quantitative (Ecological)	2005	75 Census tracts	Statewide	Estimated cancer risk due to air pollutants	n/a	1
Jephcote et al. [65]	Quantitative (Cross-sectional)	2011–2015	188,075 residents	12 parishes along Louisiana’s Mississippi River corridor ("Cancer Alley"). East Baton Rouge, Orleans, St. Bernard, St. Charles, Iberville, St. John the Baptist, West Baton Rouge, Ascension, St. James, Plaquemines, Lafourche, and Jefferson Parishes	Proximity to "highly polluting" industrial facilities producing benzene, toluene, ethylbenzene, and xylene compounds	Cancer	1
Jordan [66]	Qualitative	2021	Not provided	St. John the Baptist Parish	Chemical odors	Cancer, eye conditions, respiratory conditions	0
Jordan et al. [67]	Quantitative (Ecological)	2020–2021	415 industrial facilities	Statewide	PM 2.5	n/a	−1
Kang [43]	Qualitative	2019	Not provided	St. James Parish, Plaquemines Island, Baton Rouge (Istrouma), New Orleans (Bvlbancha), Bonnet Carr Spillway, Norco, Lake Pontchartrain	Chemical odors, diminished natural environment	Cancer, mental health, respiratory conditions	0
Karlitz et al. [68]	Quantitative (Ecological)	2005–2009	1,155,422 residents	18 Acadian parishes, in southwest and southcentral Louisiana: Acadia, Allen, Assumption, Avoyelles, Calcasieu, Cameron, Evangeline, Iberia, Jefferson Davis, Lafayette, Lafourche, Pointe Coupee, St. James, St. Landry, St. Martin, St. Mary, Terrebonne, Vermilion; statewide	n/a	Cancer	0
Katner [69]	Quantitative (Ecological)	1996–2011	64 parishes	Statewide	Estimated cancer risk due to air pollutants	n/a	0
Kim et al. [70]	Quantitative (Ecological)	2015–2018	Not provided	Statewide	Proximity to oil refinery (within 10 km)	Cardiovascular disease	1
Kodsup and Godebo [71]	Quantitative (Cross-sectional)	2020–2021	408,047 COVID-19 cases	Statewide	n/a	Cancer, cardiovascular disease, COVID-19, kidney disease, metabolic conditions, mortality, respiratory conditions	1
Legot et al. [72]	Quantitative (Ecological)	2002–2006	20 ZIP-codes	East Baton Rouge Parish	Number of TRI facilities, amount of releases, outcome-specific releases	Asthma, neurologic/neurodevelopmental conditions	0
Luckett et al. [73]	Quantitative (Case–control)	2001–2005	158 residents	Acadia, Evangeline, Iberia, Lafayette, St. Landry, St. Martin, St. Mary, and Vermillion Parishes	Urine cadmium concentrations (μg/g creatinine), living within one mile of a petroleum refinery	Cancer	1
Ma et al., [74]	Quantitative (Cohort)	1980–2017	18,990 participants	Not specified	Estimated annual average PM2.5 concentrations	Cancer	1
Maghfour et al. [75]	Quantitative (Ecological)	2000–2018	774 patients	Statewide	Benzene and trichloroethylene concentrations (measured in µg/m^3^), from NATA data (1996 - 2011)	Cancer	0
Nagra et al. [76]	Quantitative (Cross-sectional)	2016–2020	538 households	Area within a 2.5 km radius of the Denka Performance Elastomer plant, in St. John the Baptist Parish, Louisiana	Annual mean ambient chloroprene concentration (EPA); residential proximity to industrial facility (residing less than 1.5 km vs. 1.5–2.5 km from the plant)	Allergic conditions, cancer, cardiovascular disease, dermatologic conditions, eye conditions, general/constitutional symptoms, neurologic/neurodevelopmental conditions, respiratory conditions	0
Odera et al. [77]	Qualitative	2020	19 residents	Colfax, LA	Community member lived experiences and perceptions of industrial pollution	Cancer, dermatologic conditions, metabolic conditions, respiratory conditions	0
Perlin et al. [78]	Quantitative (Ecological)	1990	159,968 residents	11 Cancer Alley parishes: East Baton Rouge, West Baton Rouge, Iberville, Ascension, St. James, St. John the Baptist, St. Charles, Jefferson, Orleans, Plaquemines and Assumption Parishes	Residential proximity to TRI-reporting facilities	n/a	0
Perlin et al. [79]	Quantitative (Ecological)	1990	1,159,968 residents	11 Cancer Alley parishes: East Baton Rouge, West Baton Rouge, Iberville, Ascension, St. James, St. John the Baptist, St. Charles, Jefferson, Orleans, Plaquemines and Assumption (referred to in this study as "the New Orleans to Baton Rouge Corridor"). This paper also included data on the Kanawha Valley in West Virginia and Baltimore area of Maryland	Residential proximity to TRI-reporting facilities	n/a	0
Pezzullo [80]	Qualitative	2001	Not provided	Cancer Alley	Community member lived experiences of pollution	Cancer, neurologic/neurodevelopmental conditions, respiratory conditions	0
Pine and Diaz [81]	Quantitative (Cross-sectional)	1988–1993	Not provided	New Orleans, LA	n/a	Reproductive health	0
Rabito et al. [96]	Quantitative (Cross-sectional)	2016	76 participants	New Orleans, LA	Indoor residential black carbon levels (µg/m^3^)	Cardiovascular disease	1
Richmond-Bryant et al. [82]	Multi-method	2018	65 participants	Colfax, Louisiana	Quantitative: distance to facility; Qualitative: community member lived experiences of pollution	Cardiovascular disease, dermatologic conditions, metabolic conditions	0
Robinson et al. [83]	Quantitative (Ecological)	2023	Not applicable	4 Cancer Alley parishes: Iberville, Ascension, St. James, and St. John the Baptist Parishes	Sampled air concentrations of ethylene oxide	n/a	0
Simonsen et al. [84]	Quantitative (Case–control)	1970–2001	892 residents	11 Cancer Alley parishes (East Baton Rouge, West Baton Rouge, Iberville, Ascension, St. John, St. James, St. Charles, Orleans, Jefferson, St. Bernard, and Plaquemines Parishes)	Proximity (using buffer zones of 0.5 miles, 1 mile, and 2 miles) to TRI-reporting sites, petrochemical sites, and sites producing known or suspected carcinogens	Cancer	1
Singer [85]	Multi-method	2010	31 residents	Donaldsonville, Ascension Parish; statewide	Community member lived experiences of pollution	Asthma, anemia, cancer, cardiovascular disease, dermatologic conditions, gastrointestinal conditions, general/constitutional symptoms, respiratory conditions, neurologic/neurodevelopmental conditions	0
Terrell and James [86]	Quantitative (Ecological)	1996–2020	64 parishes	Statewide	PM 2.5, respiratory hazard index, immunological hazard index	COVID-19	1
Terrell and St. Julien [87]	Quantitative (Ecological)	2008–2017	750 census tracts	Statewide	Estimated cancer risk from air toxics	Cancer	1
Terrell and St. Julien [88]	Quantitative (Ecological)	2019–2021	671 census tracts	Statewide	Reported emissions of criteria pollutants (PM10, PM2.5, NOx, SO2, CO, and VOC)	n/a	1
Terrell et al. [89]	Quantitative (Ecological; Cross sectional)	2011–2020	1,101 census tracts	Statewide	Respiratory hazards	Reproductive health	1
Tsai et al. [90]	Quantitative (Ecological)	1970–1999	Not provided	Statewide	Residence in 8 Cancer Alley parishes (Ascension, East Baton Rouge, Iberville, St. Charles, St. James, St. John the Baptist, and West Baton Rouge) vs. others in LA	Mortality	−1
White et al. [91]	Quantitative (Cohort)	1980–2017	196,905 women	Not specified	Estimated annual average PM2.5 concentrations	Cancer	1
Wisniewski et al. [92]	Quantitative (Case–control)	2001–2003	1,291 emergency department visits	New Orleans	n/a	Asthma	0
Wong et al. [93]	Quantitative (Case–control)	2002	386 residents	Calcasieu and Lafayette Parishes	Blood serum dioxin concentrations	n/a	1
Wong et al. [94]	Quantitative (Case–control)	2002	387 residents	Calcasieu and Lafayette Parishes	Blood serum PCB concentrations	n/a	1
Yu et al. [95]	Quantitative (Cross-sectional)	2020–2021	11,331 patients	Washington, Ascension, Assumption, East Baton Rouge, Livingston, Lafayette, and Ouachita Parishes	Census tract level PM 2.5 and 21 HAPs from NEI data	COVID-19	1

^a^Study types: cross sectional studies measured pollution and/or health outcomes at one time point or time range. Case-control studies compare cases and non-cases. Cohort studies measured the health effects of pollution exposure over time. Ecological studies measured data for groups or areas (e.g., ZIP code or parish). Qualitative study data come from community member lived experiences. Multi-method studies utilize quantitative and qualitative data.

^b^ Sample size: If sample size was not provided in the main text or any supplemental materials, we note “Not provided.”

^c^Locations include parishes (counties in Louisiana), cities, and neighborhoods. Locations are listed how they are referenced in each study. “Statewide” is used for studies that report data at the level of the entire state of Louisiana. For studies reporting data from ”Cancer Alley,” specific parishes included are listed in the table.

^d^ Industrial sites and emissions were identified using the data source stated in each paper (for example TRI, LDEQ records, EPA datasets). Facility counts reflect reporting and coverage in those datasets. Unless otherwise stated, pollution media included ambient air, drinking or surface water, and soil, consistent with the review inclusion criteria. Distance or buffers reflect residential proximity to industrial facilities. “Within X miles” or “within X km” indicates the specified radius from the residence or study location. “Pounds released” refers to reported annual air releases from TRI facilities when used.

^e^ Abbreviations: EPA, United States Environmental Protection Agency; NPL, National Priorities List; NATA, National Air Toxics Assessment; PM_2.5_, particulate matter with aerodynamic diameter less than 2.5 µm; LA, Louisiana; TRI, Toxics Release Inventory; LDEQ, Louisiana Department of Environmental Quality; PM_10_, particulate matter with aerodynamic diameter less than 10 µm;, NO_x_, nitrogen oxides; SO_2_, sulfur dioxide; CO, carbon monoxide; VOC, volatile organic compound; PCB, polychlorinated biphenyls; HAP, hazardous air pollutant; NEI, National Emissions Inventory. PM_2.5_ concentration is measured in µg/m^3^. NATA reports modeled data, not direct measurements. N/a (not applicable) is used for studies that do not report pollution exposure. Air sampling studies report measured concentrations at the time and place of sampling.

^f^ Measures of health were grouped into the following categories: Allergic conditions, anemia, cancer, cardiovascular disease, COVID-19, dermatologic conditions, eye conditions, gastrointestinal disorders, general/constitutional symptoms, hearing condition, kidney disease, mental health, metabolic conditions, mortality, neurologic/neurodevelopmental conditions, overall health, reproductive health, respiratory conditions. N/a is used for studies that do not report health outcomes.

^g^ Study assessment score is a sum of the following components: +1 = adjusted for confounding using multivariable or causal methods; 0 = no confounder adjustment; −1 = no confounder adjustment and study authors had documented ties to the petrochemical industry. (No studies had both adjusting for confounding and authors with industry ties.)

Studies examined multiple health outcomes, including cancer (*n* = 22), asthma (*n* = 7), other respiratory conditions (e.g. COPD, cough, sore throat, sinus infection, sneezing; *n* = 13), mortality (all-cause or cause-specific; *n* = 14), and COVID-19 (*n* = 8). Other outcomes included: eye and/or skin irritation, cardiovascular disease (e.g. coronary heart disease, angina, hypertension, heart disease), neurologic and neurodevelopmental disorders (Parkinson's disease, childhood developmental disorders, autism, behavioral issues), diabetes, overweight/obesity, self-rated health, headaches, dizziness and/or lightheadedness, mental health outcomes, reproductive health outcomes (low birthweight, preterm birth, miscarriage, infertility), thyroid disease, kidney disease, edema, anemia, hearing impairment, and gastrointestinal conditions.

Study quality varied across the included studies, A total of 21 studies [47, 54, 55, 61, 62, 64, 65, 70, 71, 73, 74, 84, 86–89, 91, 93–96] explicitly adjusted for confounding using multivariable or causal methods and received a score of 1, while 29 studies [43, 44, 46, 48, 49, 51–53, 56–60, 63, 66, 68, 72, 75–77, 79–83, 85, 92] did not adjust for confounding. Studies that adjusted for confounding were all quantitative studies. Multi-method studies (*n* = 2) and qualitative studies (*n* = 7) did not adjust for confounding and did not have authors with petrochemical industry affiliations. Most studies had no petrochemical industry ties (*n* = 50), but we identified three studies identified as having industry affiliations and we gave them a score of −1 [50, 67, 90].

### Geographic Distribution of Studies

**Fig. 2:**
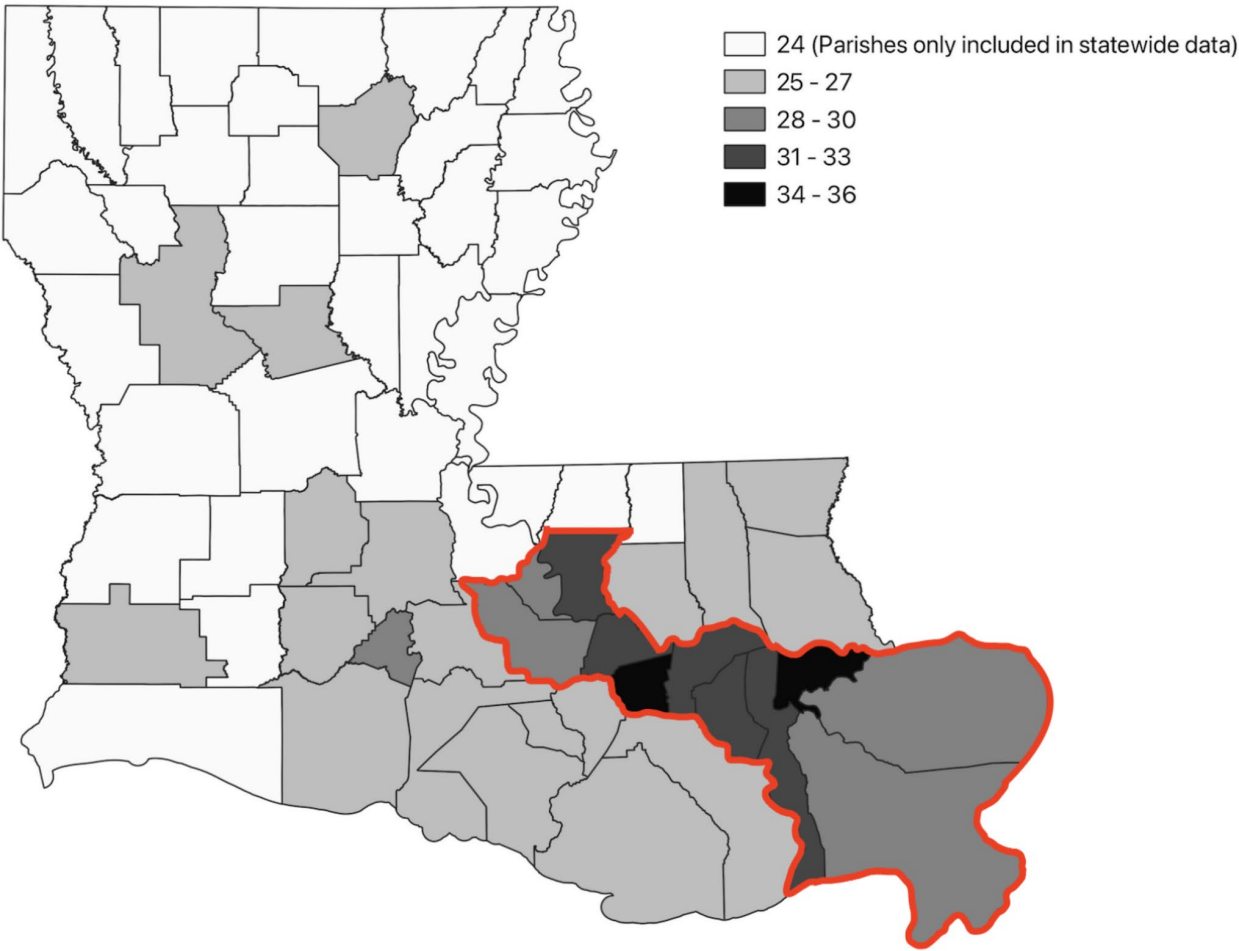
Choropleth map of number of studies reporting data on each parish. Outlined is the region known as Cancer Alley. There were 24 papers that reported on statewide data, and so technically had data from all counties

Figure 2 shows the distribution of study samples across the state. Some studies reported the geographic distribution of pollution and/or health outcomes across the entire state (*n* = 19) [47, 50, 55, 56, 58–62, 67, 70, 71, 74, 75, 86, 87, 89–91, 97], some studies limited their samples to include most or all of the Cancer Alley parishes (*n* = 6) [63, 64, 78, 79, 83, 84], while others focused on specific parishes, cities, or smaller communities within Cancer Alley and/or other industrialized areas (*n* = 25) [43, 44, 46, 48, 49, 51–54, 57, 65, 66, 72, 73, 76, 77, 80–82, 85, 92–96]. For simplicity, study locations are aggregated at the parish level in Fig. 2.

### Industrial Pollution Exposure in Louisiana

The most frequently described pollution type was air pollution. All quantitative and qualitative studies that assessed industrial pollution examined air pollution (37 quantitative, 7 qualitative, 2 multi-method), with some also assessing water pollution (8 quantitative, 4 qualitative, 1 multi-method). The most commonly used methods of measuring pollution in the quantitative studies were air quality measures (24 studies) and distance-based measures of proximity to one or more industrial facilities (13 studies). Several of these papers used EPA Toxics Release Inventory (TRI) data [54, 61, 65, 69, 72, 78, 79, 84, 98], which includes facility-reported air pollutant emissions from certain facilities in certain industries [99], while others collected their own data [73, 83, 96] or used data from other federal or state government databases, including the National Air Toxics Assessment (NATA) and the National Emissions Inventory (NEI) [64, 87, 95].

Seven studies compared Cancer Alley parishes to the rest of the state or the state as a whole [50, 58, 59, 64, 85, 86, 90]. Most studies assessed pollution and/or health outcomes in the eleven parish area along the lower Mississippi River often called “Cancer Alley” or “the Industrial Corridor” (Ascension, East Baton Rouge, Iberville, Jefferson, Orleans, Plaquemines, St. Bernard, St. Charles, St. James, St. John the Baptist, and West Baton Rouge Parishes) [50, 58, 59, 61, 63, 78, 79, 84]. Three studies defined the region slightly differently [50, 65, 90].

### Exposure to Air Pollution

Over multiple decades, researchers have documented elevated levels of industrial pollution exposure in Louisiana, and particularly in Cancer Alley. Per 1990 TRI data, the Cancer Alley region of Louisiana had a similar number of TRI-reporting facilities as the Baltimore metro area, but had over 60,000,000 pounds per year more total air releases, including over 12,000,000 pounds per year more hazardous air pollutants released; additionally, 65% of Cancer Alley residents lived within 2 miles of 2 + TRI-reporting facilities [78]. In 2023, mobile air sampling revealed that much of Cancer Alley had ethylene oxide concentrations that not only exceeded EPA’s acceptable levels but were also much higher than levels reported by the US EPA’s Air Toxics Screening Assessment [83].

In addition to elevated pollution across Cancer Alley, multiple studies across decades found that, on average, Louisiana communities with more people of color and/or lower incomes lived closer to industrial facilities and were exposed to higher levels of pollution. In 1990, there was a larger percentage of Black residents living in Cancer Alley than in Louisiana overall. Black residents and residents who lived below the poverty line in Cancer Alley were statistically significantly more likely to live closer to the nearest TRI facility [78], and Black residents below the poverty line on average lived closer to the nearest TRI facility [79]. In Baton Rouge in 1996, Black households resided closer to the nearest TRI facilities than white households, had a higher number of such facilities within three miles of their homes, and faced greater exposure to airborne toxic substances, even after controlling for income, education, and other demographic factors [54]. In 1996, New Orleans metropolitan area residents in the most polluted community with EPA National Priority List (NPL) sites—sites that have known or potential releases of pollutants, contaminants, or hazardous waste [100]—were mostly Black, low to middle income, and perceived air pollution as a serious problem compared to residents living in non-NPL sites [46]. In St. James Parish, a mostly rural parish in Cancer Alley, analyses of 1987–2002 Louisiana Department of Environmental Quality (LDEQ) data showed that the three census tracts that had the highest proportion of Black residents, and the lowest average income, hosted most of the pollutant-emitting facilities in the parish, and the facility that had the highest pollutant emissions was in the census tract with the highest proportion of Black residents and the lowest average income [52]. In East Baton Rouge Parish in 2002, the mean percentage of residents of color and those living below the poverty line were higher in both the two most polluted ZIP codes and the six ZIP codes nearest to the parish’s highest-volume polluters versus the other parish ZIP codes [72]. In 2018, within a 2.5 km radius of the Denka Performance Elastomer Plant in St. John the Baptist Parish, a higher proportion of residents living closer to the facility were Black [76]. In a study using 2019–2021 data from the LDEQ, the proportion of people of color in a census tract in Louisiana was significantly associated with higher industrial pollutant emissions, with communities of color facing 7-fold to 21-fold higher emissions than predominantly white communities [97].

### Estimated Health Risks Based on Air Pollution

Some studies examined patterns in estimated health risks based on air pollution in Louisiana. Per 2005 EPA National Air Toxics Assessment (NATA) data, estimated cancer risk from air toxics emitted from point sources was elevated in southwest Louisiana and in Cancer Alley census tracts [87], and estimated cumulative cancer risk from pollution from both stationary and mobile sources in Cancer Alley were significantly higher than the rest of the state and nationally [64]. In 2014, pollution burdens due to PM_2.5_, respiratory hazards, and immunological hazards were higher in Louisiana census tracts with more Black and unemployed residents [86]. The majority of the facilities that polluted the highest amounts of PM_2.5_ in 2020 (per EPA records) were concentrated in Cancer Alley [67].

Using data from the EPA's Risk Screening Environmental Indicators model, based on TRI data from 1996–2011, pollution (particularly chromium, polycyclic aromatic compounds, and 1,3-butadiene) in Caddo, St. John the Baptist, East Baton Rouge and Calcasieu Parishes, which are all in highly industrialized regions with numerous TRI facilities, contributed the most to statewide cancer-specific (prostate, lung, bladder, kidney, breast, and non-Hodgkin lymphoma) risk scores [69].

### Other Pollution Exposures

Two articles compared 2002 blood serum levels of certain industrial chemical byproducts among residents in two Louisiana parishes, where most residents were white and living above the poverty line: Calcasieu Parish, which had more industrial facilities, and Lafayette Parish, which had fewer [93, 94]. No significant differences were found between the two parishes or in comparison to a representative sample of the United States. Serum concentrations of the two byproducts were on average higher among Black residents in Lafayette Parish than white residents in Lafayette and both white and Black residents in Calcasieu [93, 94].

In Ascension and St. James Parishes, per 2010 Census data and 2020 zoning and land use data, Black cemeteries were more likely to be located near industrial facilities than white cemeteries. All cemeteries within 2 miles of an industrial facility were Black cemeteries [51].

### Qualitative Research on Exposure to Multiple Types of Industrial Pollution

Qualitative studies documenting Louisiana residents’ experiences of industrial pollution exposure contextualize these quantitative findings. In 2009 in Donaldsonville, Louisiana—a predominantly low-income, Black community in Cancer Alley—residents reported strong ammonia odors and linked pollution from local plants to environmental degradation, including a decline in trees and bird populations following industry's arrival [85]. In 2015–2018, residents of Freetown, St. James Parish—a predominantly Black, low-income rural community founded by former slaves in Louisiana’s Cancer Alley— reported constant exposure to strong chemical odors and loud noises from surrounding industrial facilities [44]. Residents also reported environmental degradation following the arrival of industrial facilities, including dying vegetation, stunted garden growth, and the disappearance of birds, frogs, and other wildlife. Black residents noted that while white residents were “bought out” by petrochemical companies, Black residents were left to remain in polluted areas [57]. In 2018, residents of Colfax, Louisiana, a town where 58% of residents are Black and 35% live in poverty [101, 102], described a range of environmental and health concerns related to a nearby hazardous waste treatment facility, including smoke, chemical smells, loud noise, vibrational damage to homes, illness or death of animals, difficulty growing plants, and fears about the safety of local fishing [82]. Another study interviewed residents of Colfax, Louisiana, in 2020, and residents again reported dark smoke plumes and chemical odors, loud noises, and vibrations that rattled their homes during the waste treatment facility's burning activities. They also expressed concerns about water safety and the potential contamination of local waterways, which they believed caused the decline of wildlife and vegetation [77]. In 2019, a study author observed chemical odors, orange-colored foam on and plastic debris in the Mississippi River, and "dense, toxic mud" from a spillway containing petroleum runoff in Louisiana's Lower Mississippi region [43].

### Spatial Distribution of Health Outcomes in Louisiana

#### Cancer

Five papers looked at spatial distribution of cancers in Louisiana. A study of lung tumor specimens from a population-based case–control study of individuals diagnosed 1998–2001 in the 11 Cancer Alley parishes found that certain *KRAS* mutations were more common in that sample than globally, but no associations were found with smoking or other occupational, environmental, or residential exposures [63]. A study of 2005–2008 Louisiana Tumor Registry data and 2005–2009 US Surveillance, Epidemiology, and End Results (SEER) data found significantly higher age-adjusted colorectal cancer rates in Louisiana’s 18 Acadian parishes (in southwestern Louisiana) compared to both state and national rates. Rates were highest in parishes that had more residents of likely Cajun descent (operationalized as speaking French at home) [68].

Another paper reported on three Louisiana Department of Public Health (LDPH) cancer cluster investigations in different parts of Cancer Alley using data from the Louisiana Tumor Registry. Using 1988–2004 data, cancer rates in the Livonia Fordoche area were lower than expected for men and similar to the state average for women. Using 1988–2010 data, the cancer incidence in Mossville was lower than the statewide rate. Using 1986–1996 data, there were elevated risk ratios for children’s diagnosis of acute lymphoblastic leukemia in Coteau compared to New Orleans, but a case–control study of potential risk factors was inconclusive [60].

Per Louisiana SEER data of women diagnosed with breast cancer between 2000 and 2013, parishes with shorter survival times were lower income and/or located along the Mississippi River (including but not limited to the Cancer Alley parishes) and the Red River and/or experienced significantly higher exposure to ammonia and particulate matter emissions, potentially associated with industrial and agricultural sources [55]. Using 2011–2015 National Cancer Institute data, annual age-adjusted leukemia incidence in 9 Cancer Alley parishes (rates in the other three were censored due to low case counts) did not significantly differ from the statewide rate, except in Orleans Parish, where the rate was significantly lower. Jefferson and East Baton Rouge Parishes had significantly higher 5-year case counts than the other ten parishes. New leukemia cases attributed to petrochemical exposure were spread across parishes, with the largest contributions from East Baton Rouge Parish and Orleans Parish [65].

#### Asthma

Two studies presented data about the geographic distribution of asthma in Louisiana. In 1998–1999, there were 6,498 visits to the Leonard J Chabert Medical Center, located in Houma, Terrebonne Parish, where asthma was the primary diagnosis, with a mean of 270.8 admissions per month [53]. In another study, a total of 1,291 emergency department visits for asthma exacerbations among children 6–10 years of age were recorded from September 2001 through August 2003 at the Medical Center of Louisiana in New Orleans [92].

#### COVID-19

Spatial analysis of 2020 COVID-19 data revealed early COVID-19 mortality hot spots in urban parishes (Orleans, Jefferson, East Baton Rouge) that were part of the Cancer Alley region. Subsequent COVID-19 surges affected rural parishes like East Carroll, which reported both the highest poverty rate and the highest COVID-19 mortality rate in the state [61].

#### Other Health Outcomes

From 1988 to 1993, the incidence of premature birth, as reported by the LDPH, varied across New Orleans census tracts and was higher in low-income census tracts [81].

Analysis of hospital discharge data from 1995 to 2014 identified southwestern and southeastern Louisiana as regions with higher incidences of Parkinson’s disease [62].

### Industrial Pollution Exposure and Human Health

#### Cancer

Two studies assessed the relationship between industrial pollution and cancer using NATA data for air pollution and Louisiana Tumor Registry data for cancer. In low-income Census tracts in Louisiana, higher toxic air pollution (per 2005 NATA data) was statistically significantly correlated with increased cancer rates (per 2008–2017 Louisiana Tumor registry data), but not in higher-income Census tracts; neither smoking nor obesity rates explained the relationships [87]. Using data from the Louisiana Tumor Registry (2000–2018) and the EPA’s NATA database (1996–2011), there was no significant relationship between cutaneous T-cell lymphoma incidence and exposure to either benzene or trichloroethylene in a multivariable analysis, but one of the two higher-incidence areas (southeastern Louisiana) also had the highest benzene and trichloroethylene exposure levels in the state [75].

Two studies assessed the association between PM_2.5_ and cancers in the NIH-AARP cohort. There was no association between long-term exposure to PM_2.5_ (estimated using EPA data and spatiotemporal prediction models) and incident liver [74] or breast [91] cancer among Louisianans in this cohort. A significant association was found among the entire cohort for breast cancer, but Louisianans were only ~ 4% of the total cohort [91].

A survey conducted in 2018 of local residents living near the Denka Performance Elastomer Plant in St. John Parish, Louisiana (a predominantly Black, low-income area located in Cancer Alley), found that those residing within 1.5 km of the plant had a significantly higher than expected cancer prevalence, while those living between 1.5 km and 2.5 km from the plant did not have a significantly different prevalence than expected [76].

Two case–control studies considered residential proximity to petrochemical facilities and cancer. A study using data from 1970–1997 in Cancer Alley found that, after adjusting for potential confounders, the odds ratios for lung cancer for those living within a half-mile of any active industrial facility or petrochemical facility were not statistically significant [84]. A case–control study of pancreatic cancer cases diagnosed in eight parishes in south Louisiana hospitals from 2001–2005 and controls randomly selected from government databases found that self-reported history of living within one mile of an oil refinery was associated with an increase of urinary cadmium concentration, and that increasing urine concentrations of cadmium were statistically significantly associated with increased pancreatic cancer risk, but the direct association between living near a refinery and pancreatic cancer was not statistically significant [73].

#### COVID-19

Eight studies highlighted significant geographic disparities in COVID-19 infection, hospitalization, and mortality in Louisiana, particularly in the industrialized Cancer Alley region. During the first half of 2020, in six of the Cancer Alley parishes, COVID-19 death rates were 2.1–3.7 times higher than the state median [86], and in the 11 Cancer Alley parishes, the risk of COVID-19 infection and mortality was 1.71 times and 2.62 times higher, respectively, than the rest of Louisiana [59]. In Cancer Alley, Black residents experienced a higher mortality risk than white residents across the nine parishes with available race-stratified data [59] and in Ascension and West Baton Rouge Parishes—both heavily industrialized areas within the region [58]. Residence in Cancer Alley parishes was correlated with both COVID-19 cases and deaths in 2020 [59] and 2021 [56].

Across Louisiana, higher COVID-19 death rates correlated with greater air pollution burdens and higher percentages of Black residents, independent of diabetes, obesity, or smoking [86]. A study that analyzed census-tract level EPA air pollution data from 2014 and 2016, CDC’s 2018 overall social vulnerability data, and cumulative COVID incidence from 2020–2021 from LDPH data found moderate correlations between census tracts with higher ozone levels and cumulative COVID-19 incidence from March-June 2020 [47].

In a study using 2020 COVID-19 infection data in Louisiana, parishes with higher levels of environmental hazards (e.g., toxic releases, air pollution, drinking water violations, and lack of green space) also had high COVID-19 mortality rates [61]. Another study using a local health system’s COVID-19 registry in Cancer Alley in 2020–2021 found that Black patients experienced disproportionately high rates of COVID-19 diagnosis, hospitalization, ICU admission, and death relative to their share of the patient population, and that exposure to certain air pollutants, including chlorine, PM_2.5_, and hydrochloric acid, may partly explain the racial disparities in COVID-19 outcomes observed [95]. Similarly, parishes with a ≥ 40% Black population in Cancer Alley had the highest COVID-19 case and death counts from March 2021-March 2022 [71].

#### Mortality

Several studies assessed mortality; those focused on mortality related to cancer and COVID-19 are discussed above. In 2009 in Donaldsonville, Louisiana, a predominantly low-income, Black community within Cancer Alley with strong chemical industrial facility presence, among deaths processed by a mortuary that processed approximately half of all Black individuals buried, 17.5% of Black residents whose deaths were processed by the mortuary died of cancer, with an average lifespan of 57.5 years—far below the U.S. average life expectancy of 78 years for Black people. Those who died of other causes had an average lifespan of 61.2 years [85]. One study assessed premature death (years not indicated and not provided by authors upon request, but likely 2017–2019) by parish, and found that years of potential life lost (YPLL) was 40% higher in the eleven parishes in Cancer Alley compared to the U.S, and that St. Bernard Parish in particular had a YPLL 70% greater than the US average. More than half of Cancer Alley parishes had higher YPLL than the rest of Louisiana [58].

#### Self-Rated Health

Three studies assessed self-rated health in communities that experienced pollution. In Baton Rouge in 1996, where Black residents experienced higher exposure to air pollution than white residents, Black participants were more likely to rate their health as "fair" or "poor" than white participants [54]. In the New Orleans metropolitan area in 1996, residents of a polluted community perceived their health and wellbeing to be significantly worse than those who did not live in the polluted community [46]. In 2017, the percent of residents in Cancer Alley parishes who reported their health as fair or poor (18–25%) was similar to that for Louisiana as a whole (21%) (data source not indicated and not provided by authors upon request). The numbers of self-reported physically unhealthy days and mentally unhealthy days were also similar in Cancer Alley to the state as a whole [58].

#### Cardiovascular Health

Three studies assessed associations between pollution and cardiovascular health in Louisiana. In addition to reporting increased COVID-19 exposure and mortality rates associated with parishes with increased environmental hazards, Hu et al. [61] also reported greater cumulative health risks, including higher rates of obesity, diabetes, and stroke [61]. In a study that used 2018 CDC PLACES survey data, 1.5% (95% CI: 0.2%−2.8%) of self-reported coronary artery disease cases were estimated to be attributable to residential exposure to oil refineries among Louisiana residents living within 5 km of a refinery [70]. Another study found that, among New Orleans residents in 2016, higher short-term indoor black carbon concentration (measured using continuous air monitoring in participants’ homes) was significantly associated with higher systolic blood pressure after controlling for confounding, and stronger effects were observed among participants with pre-existing hypertension [96].

#### Birth Outcomes

One study assessed the association of air pollution and birth outcomes in Louisiana using data from 2010–2020, and found that air pollution (measured as respiratory hazard, from 2017 EPA data) was significantly associated with low birthweight and preterm birth in Louisiana census tracts. Residents of the most polluted census tracts had a 36% higher risk of low birthweight and a 25% higher risk of preterm birth than the least polluted tracts. An average of 2,166 cases of low birthweight and 3,583 cases of preterm birth were attributed to pollution exposure annually [89].

#### Asthma

Three studies assessed pollution and asthma. Using 2002–2006 hospital inpatient discharge data for asthma from the Louisiana Department of Health and Hospitals and 2011 data from the Louisiana Department of Education on child neurodevelopmental disorders, in East Baton Rouge Parish, average rates of asthma were 1.6 times higher, and child disability was 1.39 times higher in the two most polluted ZIP codes (where 18 of the 24 TRI-reporting facilities in the parish were located in 2002), compared to all other ZIP codes in the parish [72]. In Louisiana, higher ozone levels (per 2016 NATA data) were correlated with both asthma hospitalizations and asthma prevalence at the census tract-level [47]. Louisiana parishes with high levels of environmental hazard (measured using the EPA’s Environmental Quality Index (EQI)), also had higher cumulative health risks, including asthma [61].

#### Other Health Outcomes

##### Quantitative Research

In 1996 in the New Orleans metropolitan area, residents of Agriculture Street, an EPA National Priority List (NPL) site (sites that have known or potential releases of pollutants, contaminants, or hazardous waste [100]) reported experiencing several health outcomes significantly more frequently than communities not near NPL sites, including breast cancer and spontaneous abortion [46].

In August 2012, local residents were interviewed after a chemical release occurred at the Sun Drilling chemical facility in Belle Chasse; 41% reported respiratory irritation, 37% reported headaches, and 18% reported eye irritation. After an accidental chemical release incident in 2012 at the Shell Motiva Refinery in Norco, Louisiana, 21% of local residents interviewed reported respiratory irritation, 21% reported headaches, and 18% reported eye irritation. In 2013, following elevated sulfur dioxide levels near the Chalmette Refinery, in Chalmette, Louisiana, 53% of residents interviewed reported experiencing health effects (36% reported respiratory irritation, 29% headaches, and 23% eye irritation, among other issues); nine sought medical attention [48].

Two studies analyzed residential proximity to stationary sources of industrial pollution and a variety of health outcomes. A 2018 survey of local residents living within a 2.5 km radius of the Denka Performance Elastomer Plant in St. John Parish, Louisiana (a predominantly Black, low-income area located in Cancer Alley) collected self-reported prevalence of various chronic and acute health outcomes among residents within the 1.5 km and 2.5 km buffer zones around the facility, and found most health outcomes, including chest pain, difficulty breathing, headaches (in both adults and children), dizziness, cough, and fatigue, were more prevalent in the < 1.5 km zone [76]. In 2018, in Colfax, Louisiana, residents living near a hazardous waste thermal treatment facility described experiences with thyroid, respiratory, and skin effects, among other health issues, with most affected individuals residing within a five kilometer radius of the facility and some outside of this radius but within the path of the plant’s emissions, based on the study’s emissions dispersion modeling using LDEQ data. Analyses of materials processed at the facilities revealed compounds linked to effects on the skin, eyes, respiratory system, gastrointestinal tract, cardiovascular health, and thyroid, with some mixed evidence regarding cancer risk [82].

##### Qualitative Research

Several qualitative studies highlighted the health concerns of residents of fenceline communities in Louisiana related to industrial pollution exposure. A study using media archives and interviews of local residents and activists from 1991–2001 highlighted the controversy around a proposed industrial facility in St. James Parish, Louisiana, a predominantly Black community already burdened by significant industrial pollution. Residents described experiencing frequent illness and deaths, including cancer diagnoses and child illnesses such as asthma, and posited that these illnesses and deaths were caused by pollution from industry in their community [49]. Another paper described the author’s experience on a Sierra Club-sponsored “toxic tour” of Cancer Alley in 2001. The tour guide and local residents shared personal accounts of illnesses that they attributed to industry pollution, including respiratory problems, cancer, and learning disabilities. As one local resident stated, “You can get a job, but only if you're willing to do work that will harm you, your families, and your neighbors” [80]. Residents of Donaldsonville—an economically struggling, predominantly Black town located within Cancer Alley—were interviewed in 2010 about health and environmental concerns. Residents described noticing chemical odors and linked health issues such as cancer, diabetes, high blood pressure, asthma, heart problems, miscarriages, and nausea to air and water pollution from 14 major industrial plants in the area, as well as other sources (sugar cane field burning and a local dump site) [85]. In Freetown, Louisiana, a majority Black community surrounded by a dense concentration of petrochemical plants and industrial infrastructure, Black residents interviewed in 2016–2018 reported experiencing varied health outcomes, including skin irritation, dizziness, sinus infections, headaches, cancer, diabetes, heart failure, and respiratory problems, which they attributed to the chemical pollution from the many surrounding petrochemical plants [57]. During Kang’s travels through Cancer Alley, he also notes residents linking their poor health outcomes to the proximity of industrial facilities [43]. In 2020, a study interviewed residents of Colfax, Louisiana living near a hazardous waste thermal treatment facility; participants reported skin issues, hair loss, thyroid problems, cancer, respiratory issues, and heart conditions. Community members expressed frustration over the impact on their quality of life, noting the loss of clean air [77]. Residents living near an industrial facility in St. John the Baptist Parish interviewed in 2021 reported experiencing extreme chemical odors, eye and sinus irritation, coughing, cancer cases, and “rare health conditions”, which they believed to be linked to high chloroprene emissions from the plant [66].

#### Quantitative Analyses with Industry Connections

Three papers had documented industry ties. One paper, in which the first author and two other authors reported being employed by the Shell Oil Company, analyzed data from the University of Pittsburgh's Mortality and Population Data System for 1970–1999. All-cause and all-cancer mortality was generally higher in the Industrial Corridor (same geographic region others described as Cancer Alley) and in Louisiana than in the rest of the United States for all three time periods, and non-whites had higher mortality rates across most causes. All-cause mortality for white people was significantly lower in the Industrial Corridor than in all of Louisiana; non-white people had slightly higher all-cause mortality in the Industrial Corridor than in all of Louisiana, but this difference was not statistically significant. For cause-specific mortality, white people had either no significant difference or had significantly lower mortality in the Corridor than in Louisiana [90]. An analysis of Louisiana Tumor Registry data from 1997–2001 that acknowledged a member of the Louisiana Chemical Association (a business association of chemical manufacturers in the state) examined incidence of common cancers in the Industrial Corridor (seven of the eleven parishes typically defined as Cancer Alley) and reported higher prostate cancer incidence for both Black and white men and lower lung cancer incidence for Black women and white men in those 7 parishes than Louisiana as a whole, and no significant differences in breast and colorectal cancer incidence between Cancer Alley and the state for any race-gender group [50]. A study modeling estimated PM_2.5_ reductions from industrial sources with carbon capture and storage was authored by employees of Carbon Solutions LLC, a private consulting, research and software company focused on carbon capture and decarbonization. The company provides their services to the government and industry; however, this study was only funded from government sources [67].

## Discussion

Studies in this systematic review provide evidence of geographic differences in environmental pollution and health in Louisiana. On average, residents who experienced more industrial pollution often experienced worse health, with residents of Cancer Alley bearing a disproportionate burden of environmental pollution and related health consequences. Across Louisiana, many studies found that Black and/or low-income residents were disproportionately exposed to industrial pollution. Quantitative studies identified high levels of industrial pollution, particularly air pollution, in heavily industrialized areas, but findings on the association between pollution and health outcomes were mixed. Some studies found higher rates of cancer, asthma, developmental disorders, COVID-19-related outcomes, and adverse birth outcomes in polluted areas [46, 47, 54–56, 61, 72, 76, 86, 87, 89, 95], while others had null results, potentially due to lack of statistical power and/or industrial ties [50, 73, 75, 84, 90]. Qualitative studies reported that fenceline communities experienced smoke plumes, chemical odors, environmental degradation, and elevated rates of illnesses, which residents attributed to industrial pollution.

### Discussion of Findings

Studies reviewed identified a wide range of acute and chronic health outcomes that could be linked to industrial pollution. Acute health issues included headaches, dizziness, fatigue, shortness of breath, chest pain, irritation of the eyes, nose, throat, and skin, and COVID-19 infection and severity. Chronic health issues included respiratory and heart problems, thyroid disease, cancers, and pregnancy-related outcomes. Most studies focused on adult health; there was only one study that focused on children.

Several papers focused specifically on assessing links between industrial pollution and health, with mixed results. Many found evidence of higher estimated cancer risk in industrialized areas, with the highest burden among Black and low-income people. Some studies identified statistically significant associations between industrial pollution and various health outcomes, including cancer, respiratory illnesses, and adverse birth outcomes, reinforcing community member concerns documented in qualitative studies in our review. These findings align with previous reviews. A 2022 review of 45 studies found that exposure to industrial pollutants– such as PM_2.5_, benzene, cadmium, and mixtures of industrial pollutants– as well as living near an industrial area, was associated with worse birth outcomes [103]. A 2025 review showed that communities closer to petrochemical facilities experienced higher concentrations of air pollutants like volatile organic compounds, sulfur dioxide, nitrogen oxides, and particulate matter, all of which are harmful to human health; internationally, residents of communities near petrochemical facilities had elevated risks of poor health outcomes, including cancers, chronic respiratory conditions like asthma, and reproductive issues, as seen in some studies in our review [104]. Conversely, other papers in our review reported null findings, possibly due to limitations in exposure assessment or small sample sizes that reduced the statistical power needed to detect associations, as seen in a study with limited statistical power for state-specific hazard ratios [91]. Two out of the three studies with a methodological quality score of −1 (industry ties), had null findings [50, 90]. And, these two studies had authors with stronger ties to the petrochemical industry, specifically, the Louisiana Chemical Association and the Shell Oil Company.

Another review of 36 studies around the world found no consistent association between pollution exposure and childhood asthma-related outcomes, although the authors highlighted substantial heterogeneity in study designs and methodological limitations, particularly in exposure assessment [105]. We observed comparable issues in several of the papers included in our review.

### Measures of Environment and Health

Many studies in this review relied on government pollution data, such as EPA TRI, EPA NATA, and LDEQ databases. These data are based on self-reported emissions from industrial facilities rather than direct measurements of pollutant concentrations in affected communities, which may lead to underestimates of true pollution levels, as Robinson et al. found [83]. Similar discrepancies have been documented in other studies across the U.S [83]. Additional data sources, including independent monitoring and qualitative accounts, can provide a more complete picture of exposure, but are often considered less credible. Studies in the review also had significant differences in spatial analysis methods, such as assessing pollution at the county or census tract level or using distance-based buffers around pollution sources, which could influence study findings through exposure misclassification. Additionally, using more nuanced pollution exposure estimation, such as incorporating refinery production and wind patterns, may strengthen associations with health outcomes, as shown in a study where adjusting for these factors revealed a significant link between oil refinery proximity and coronary heart disease [70].

Studies varied in how they measured health outcomes, with some using self-reported data from surveys, while others used government health data or hospital records. Self-reported data allows for broader inclusion of conditions that might not be captured in medical records, but is subject to recall bias; in contrast, hospital discharge data, cancer registries, or other government data may have more objective measures but may miss those without consistent access to healthcare or those with undiagnosed conditions. These differences in measurement could contribute to variations in study results.

### Strengths

To our knowledge, this is the first systematic review of research about industrial pollution and health in Louisiana, a state known for its environmental pollution issues. Our review had several strengths. We used a comprehensive search strategy in multiple databases, and used a two-reviewer method to ensure accuracy and consistency in line with PRISMA guidelines. Our review fills a knowledge gap in that it synthesizes qualitative and quantitative studies to provide a holistic view of this topic.

### Limitations

Despite our comprehensive approach, this review is limited by the heterogeneity of exposure and outcome assessment methods, which precluded meta-analysis and can have their own limitations, and predominance of cross-sectional designs among quantitative studies, which limit causal inference. While the inclusion of a variety of types of studies in our review is a strength, it also introduces a limitation, in that methodological differences between studies can make comparisons difficult. This systematic review is also affected by publication bias: it is well-established that, for quantitative studies, manuscripts reporting on statistically significant findings are more likely to be published.

The methodological quality scoring system we used is a simplified metric that does not fully capture study quality or risk of bias. For example, qualitative studies cannot adjust for confounding in their findings, which limits the study assessment score of qualitative studies included in this review. And, although some studies adjusted for confounders, there may still be unmeasured confounding present. We were able to find a pattern of industry connections among null studies, but it is difficult to assess patterns in the studies with greater quality scores given the broad range of findings reported.

### Directions for Future Research

We noted that many studies in our systematic review only considered environmental patterns or only considered health patterns, or only had cross-sectional data. We recommend that future researchers conduct studies that include both environmental and health data, ideally longitudinally, to support assessments of temporality and potential causality. Additionally, there may also be opportunities to use quasi-experimental analyses or other approaches that may support causal inferences, including assessing the impacts of changes in permitting, policies, and/or other regulations.

### Implications for Policy and Practice

There are multiple implications for policy and practice. These studies document a long history of environmental and health issues, as well as substantial evidence of environmental and health burdens disproportionately affecting historically marginalized communities. Considering cumulative health impacts in zoning and permitting processes may be one potential public health intervention, as well as providing additional health resources to communities experiencing higher health burdens.

## Conclusion

This systematic review of 53 articles highlights key findings, including substantial quantitative and qualitative evidence of elevated exposure to air pollution in industrialized regions of Louisiana, worse health outcomes in communities with higher industrial exposures, and links between industrial pollution and health in Louisiana. Communities experiencing more environmental pollution and worse health also were, on average, of lower-socioeconomic position and had larger proportions of Black residents. There is a long history of documenting environmental and health issues in Louisiana, and substantial evidence exists that can inform environmental planning and public health decision-making.

## Data Availability

No datasets were generated or analysed during the current study.
